# The relationship between serum uric acid within the normal range and β-cell function in Chinese patients with type 2 diabetes: differences by body mass index and gender

**DOI:** 10.7717/peerj.6666

**Published:** 2019-03-26

**Authors:** Xing Zhong, Deyuan Zhang, Lina Yang, Yijun Du, Tianrong Pan

**Affiliations:** Department of Endocrinology, The Second Affiliated Hospital of Anhui Medical University, He Fei, Anhui Province, China

**Keywords:** Serum uric acid, β-Cell function, Overweight, Obesity

## Abstract

**Background:**

Elevated serum uric acid (SUA) has a positive correlation with insulin secretion and insulin resistance indexes. However, whether weight- and gender-specific differences regarding the relationship between SUA within the normal range and β-cell function and insulin resistance exist is unknown in type 2 diabetes mellitus (T2DM) patients.

**Methods:**

A total of 380 patients with type 2 diabetes were divided into two groups as overweight/obesity (*n* = 268) and normal weight (*n* = 112). Each group were again divided into low (LSUA) and high normal SUA (HSUA). The HbA1c, C-peptide, SUA, creatinine, and lipids profiles were measured. HOMA2IR and HOMA%2B were estimated using fasting glucose and C-peptide by homeostasis model assessment (HOMA). Pearson’s correlations and multiple linear regression analyses were conducted to assess the associations between SUA levels and islet function indexes.

**Results:**

In overweight/obesity subgroup, the levels of body mass index, fasting C-peptide (FCP), P2hCP, fasting CPI (FCPI), postprandial CPI (PPCPI), ΔC-peptide, HOMA2%B, and HOMA2IR were higher in HSUA group than in LSUA group. In contrast, the HbA1c, FBS, and P2hBS were lower in HSUA than in LSUA. In normal weight subgroup, there were no differences between the HSUA than LSUA group in terms of clinical characteristics. Pearson’s correlations indicated that there were no significant correlations between SUA and insulin secretory capacity in normal weight group, but in overweight/obesity group, SUA had positive significant correlations with P2hCP, FCPI, PPCPI, ΔC-peptide, and HOMA2%B. In the female group, there were no significant correlations between SUA and insulin secretory capacity. However, in the male group, SUA had positive significant correlations with insulin secretory capacity include P2hCP, FCPI, PPCPI, ΔC-peptide, and HOMA2%B. Multiple linear regression showed that SUA was significantly associated with HOMA2%B, but not with HOMA2IR in overweight/obesity and male group.

**Conclusions:**

Our study shows that SUA levels within normal range were associated with β-cell function in T2DM patients with overweight/obesity or male. This finding supports that the association between SUA within normal range and insulin secretion ability differs by weight and sex.

## Introduction

Type 2 diabetes mellitus (T2DM) has become a serious issue in China with increasing incidences over the past decades (Ogurtsova et al., 2017). Increasing evidence suggests that high serum uric acid (SUA) level is not only associated with metabolic syndrome (MS) (Babio et al., 2015), but also is regarded as a potential tool for early diagnosis of MS (Chen et al., 2016). Elevated the level of SUA is associated with increased risk of T2DM and prediabetes in individuals with normoglycaemia in a large population-based cohort study (Dehghan et al., 2008; Van Der Schaft et al., 2017). However, changes in SUA and blood glucose do not exhibit a linear relationship. SUA rises with increasing blood glucose concentrations in the normal and prediabetes population, while SUA levels are negatively associated with HbA1c in T2DM (Kawamoto et al., 2018).

Progressive deterioration of islet β-cell function and insulin resistance are considered as primary pathophysiological factors during the development of T2DM. SUA is the end product of an exogenous pool of pruines and endogenous purine metabolism, and the final oxidation product of purine metabolism in humans, which is responsible for the production of UA and damage of free radicals. In hyperuricemic subjects with IGT, the failure of β-cell function to compensate variation of insulin sensitivity, compared with non-hyperuricemic (Simental-Mendia, Rodriguez-Moran & Guerrero-Romero, 2009). Furthermore, elevated SUA harbors a positive correlation with insulin secretion and insulin resistance indexes in newly diagnosed T2DM patients (Hu et al., 2018), implying a possible role for SUA in β-cell function. However, the interaction of SUA within the normal range and body mass index (BMI) on β-cell function and insulin resistance in T2DM patients remains unknown.

Therefore, we investigated the relationship between SUA within the normal range and β-cell function as well as their potential confounding factors such as age, gender, diabetic duration, blood pressure, blood lipid profiles, renal function, and HbA1c by BMI and gender.

## Materials and Methods

### Study subjects

A total of 380 patients with type 2 diabetes who visited the Second Affiliated Hospital of Anhui Medical University were randomly selected in this cross-sectional study. The diagnosis of T2DM was according to the criteria of the American Diabetes Association. The exclusion criteria were (1) with hyperuricemia defined as SUA ≥420 umol/L in men and ≥360 umol/L in women (Fang & Alderman, 2000), (2) with renal dysfunction defined as serum creatinine ≥106 umol/L in male and ≥97 umol/L in female or chronic kidney disease, (3) patients with severe pancreatic disease and liver disease and those who suffered recent diabetic ketoacidosis and hyperosmotic nonketotic diabetic coma. Written informed consent was provided by all participants. The study was approved by the Ethics Committee of the Second Affiliated Hospital of Anhui Medical University (approval number 2017027).

### Measurements

Study participants were asked about their age and family history. Body weight, height, and blood pressure were measured by the diabetic nurses. BMI was calculated by dividing weight (in kilograms) by the height (in meters) squared. Normal weight and overweight/obesity were defined as BMI <24 kg/m^2^ and BMI ≥24 kg/m^2^ for Chinese population, respectively, according to the Working Group on Obesity in China’s BMI criterias (Hou et al., 2013; Zhou, 2002). Blood tests were carried out after an overnight fasting for glucose, serum total cholesterol (TC), triglycerides (TG), high-density lipoprotein cholesterol (HDL), low-density lipoprotein cholesterol (LDL), SUA, liver/renal functions and glycated hemoglobin (HbA1c).

After collecting fasting blood samples, subjects received a noodle mixed-meal in patients with T2DM. Blood samples were collected to measure the concentrations of glucose and C-peptide 2h after the meal. HOMA2IR and HOMA%2B were assessed using homeostasis model assessment based on paired of fasting plasma glucose (FPG) and FCP measurements (https://www.dtu.ox.ac.uk/homacalculator/) (Wallace, Levy & Matthews, 2004). Insulin secretory capacity was also evaluated by C-peptide index (CPI) and ΔC-peptide. Fasting CPI (FCPI) and postprandial CPI (PPCPI) were calculated by a ratio of serum C-peptide (nmol/L) to plasma glucose concentrations (mmol/L) at baseline and 2 h after meal. The value of ΔC-peptide was defined as increment in serum C-peptide level (nmol/L) at 2 h after the meal.

Serum C-peptide was measured by chemiluminescent enzyme immunoassay. HbA1c was measured by high-performance liquid chromatography. Plasma glucose was evaluated with the glucose oxidase method. TC, TG, HDL, LDL, SUA, and liver/renal functions were analyzed by the standardized enzymatic method.

### Statistical analyses

Continuous variables were expressed as means and standard deviation or medians and interquartiles. Categorical variables were expressed by numbers. In all the analyses, parameters with non-normal distributions were used after log transformation. For categorical variables, the Chi-square test was performed, while for continuous variables, Student *t*-test was used. Pearson’s correlations were calculated to characterize the associations between islet function indexes and SUA levels within each group. To evaluate whether SUA was an independent risk factor for β-cell function in T2DM, we performed the multiple linear regression analysis. A two-tailed *p* ≤ 0.05 was considered as statistically significant. All statistical analyses were conducted with SPSS software (Version 21.0).

## Results

### Clinical and laboratory data of the patients according to BMI and SUA category

The characteristic of the study patients according to BMI was shown in Table 1. The levels of SBP, DBP, TG, FCP, P2hCP, FCPI, PPCPI, HOMA2%B, and HOMA2IR were higher in the overweight/obesity group than in normal weight group. Furthermore, the patients were divided into two groups according the median SUA levels of patients with normal weight or overweight/obesity, respectively (low-normal SUA (LSUA) ≤285 umol/L; high-normal SUA (HSUA) >285 umol/L). In the overweight/obesity subgroup, the levels of BMI, ALT, CR, FCP, P2hCP, FCPI, PPCPI, ΔC-peptide, HOMA2%B, and HOMA2IR were higher in HSUA group than in LSUA group. In contrast, the HbA1c, FBS, P2hBS, and HDL were lower in HSUA than in LSUA (Table 2). In normal weight subgroup, there were on differences between the HSUA and LSUA group in terms of clinical characteristics (Table 2).

**Table 1:** Clinical characteristics and islet function indexes of T2DM patients by BMI.

Variables	Normal weight group (*N* = 112)	Overweight/obesity group (*N* = 268)	*t/ϰ*	*p*
SUA (umol/L)	262.5 (224.3, 297.0)	290.5 (256.0, 333.0)	−5.08	<0.001
Age (years)	54.1 ± 11.9	52.1 ± 12.0	1.50	0.134
Male/female	63/49	171/97	1.38	0.168
Duration (years)	5.0 (1.0, 10.0)	4.0 (0.3, 9.7)	0.51	0.613
SBP (mmHg)	120.0 (110.0, 131.5)	130.0 (120.0, 140.0)	−2.06	0.040
DBP (mmHg)	77.0 (70.0, 84.8)	80.0 (76.0, 90.0)	0.90	<0.001
BMI (kg/m^2^)	22.3 (20.6, 23.4)	26.1 (25.4, 28.2)	−21.3	<0.001
TG (mmol/L)	1.38 (0.88, 2.12)	2.00 (1.22, 3.12)	−4.24	<0.001
TCH (mmol/L)	4.37 (3.87, 5.11)	4.54 (3.91, 5.20)	−1.01	0.315
LDL (mmol/L)	2.58 (2.18, 2.93)	2.58 (2.18, 3.10)	0.39	0.697
HDL (mmol/L)	1.07 (0.84, 1.38)	1.01 (0.76, 1.10)	2.86	0.004
ALT (U/L)	18.0 (14.0, 27.0)	21.0 (15.0, 33.0)	−1.87	0.063
CR (umol/L)	68.5 (58.0, 81.8)	73.0 (62.0, 85.0)	−1.73	0.084
HbA1c (%)	9.40 (7.53, 11.20)	8.90 (7.60, 10.70)	0.86	0.391
FPG (mmol/L)	9.49 ± 3.38	9.32 ± 3.03	0.47	0.637
P2hPG (mmol/L)	19.17 ± 4.91	18.69 ± 4.37	0.95	0.344
FCP (nmol/L)	1.84 (1.31, 2.82)	2.40 (1.79, 3.31)	−4.28	<0.001
P2hCP (nmol/L)	5.03 (3.52, 7.21)	5.90 (4.13, 7.74)	−2.54	0.011
FCPI	0.22 (0.16, 0.32)	0.28 (0.19, 0.37)	−3.77	<0.001
PPCPI	1.49 (0.94, 2.35)	1.78 (1.14, 2.62)	−2.24	0.026
ΔC-peptide	2.92 (1.76, 4.68)	3.23 (1.90, 4.62)	−1.16	0.245
HOMA2%B	42.2 (28.0, 69.0)	49.7 (33.9, 78.4)	−2.39	0.017
HOMA2IR	1.66 (1.17, 2.43)	2.11 (1.60, 3.11)	0.14	<0.001

**Note:**

Values are expressed as mean ± standard deviation (SD) or median (range 25th–75th percentile).

**Table 2:** Clinical characteristics and islet function indexes of overweight/obesity and normal weight group by the median of SUA.

Variables	Overweight/obesity group				Normal weight group			
	LSUA	HSUA	*t/ϰ*	*p*	LSUA	HSUA	*t/ϰ*	*p*
SUA (umol/L)	<285	285–420			<285	285–420		
Age (years)	52.9 ± 11.2	51.4 ± 12.5	1.01	0.314	55.4 ± 10.9	51.7 ± 13.5	1.60	0.112
Male/female	62/56	109/41	11.58	0.001	38/34	25/15	0.99	0.320
Duration (years)	4.0 (0.3, 10.0)	4.0 (0.29, 9.00)	0.14	0.886	6.0 (1.0, 10.0)	4.5 (0.42, 10.0)	−0.18	0.861
SBP (mmHg)	129.4 ± 16.3	128.7 ± 17.3	0.35	0.729	126.2 ± 17.1	122.8 ± 20.0	0.96	0.345
DBP (mmHg)	80.5 ± 10.2	82.1 ± 11.6	−1.19	0.235	76.9 ± 9.5	77.1 ± 9.6	−0.07	0.953
BMI (kg/m^2^)	26.5 ± 1.9	27.4 ± 2.7	−3.14	0.002	21.8 ± 1.9	21.6 ± 2.0	−0.17	0.872
TG (mmol/L)	1.88 (1.09, 2.58)	2.08 (1.34, 3.32)	−1.42	0.156	1.21 (0.84, 2.03)	1.43 (1.00, 2.15)	−0.51	0.614
TCH (mmol/L)	4.46 (3.74, 5.35)	4.57 (4.07, 5.15)	−0.83	0.407	4.37 (3.95, 5.08)	4.33 (3.51, 5.26)	0.53	0.595
LDL (mmol/L)	2.58 (2.19, 2.95)	2.59 (2.17, 3.13)	−0.59	0.550	2.58 (2.31, 2.93)	2.58 (2.02, 3.15)	−0.37	0.712
HDL (mmol/L)	1.07 ± 0.38	0.97 ± 0.40	2.35	0.020	1.24 ± 0.49	0.99 ± 0.29	2.94	0.004
ALT (U/L)	20.0 (14.0, 30.3)	23.5 (17.0, 35.0)	−2.73	0.007	18.0 (14.3, 23.0)	20.0 (14.0, 30.0)	−0.65	0.515
CR (umol/L)	70.9 ± 16.1	75.4 ± 14.9	−2.53	0.012	70.2 ± 15.5	70.7 ± 14.8	−0.16	0.872
HbA1c (%)	9.50 ± 2.13	8.89 ± 1.96	2.40	0.020	9.32 ± 2.32	9.71 ± 2.75	−0.78	0.434
FPG (mmol/L)	9.7 ± 2.8	9.0 ± 3.2	2.16	0.032	9.5 ± 3.3	9.5 ± 3.5	0.08	0.931
P2hPG (mmol/L)	19.4 ± 3.9	18.1 ± 4.7	2.44	0.015	18.9 ± 4.9	19.5 ± 4.9	−0.49	0.636
FCP (nmol/L)	2.24 (1.71, 3.02)	2.50 (1.87, 3.41)	−2.52	0.012	1.81 (1.30, 2.74)	1.92 (1.32, 3.09)	−0.87	0.388
P2hCP (nmol/L)	5.00 (3.63, 6.73)	6.52 (4.87, 8.43)	−4.45	<0.001	4.87 (3.20, 6.68)	5.46 (3.58, 7.69)	−0.72	0.474
FCPI	0.24 (0.17, 0.34)	0.31 (0.22, 0.42)	−3.82	<0.001	0.22 (0.16, 0.30)	0.25 (0.15, 0.36)	−0.88	0.381
PPCPI	1.46 (0.95, 2.36)	2.04 (1.35, 2.95)	−4.52	<0.001	1.45 (0.94, 2.18)	1.76 (0.94, 2.60)	−0.36	0.716
ΔC-peptide	2.52 (1.44, 4.07)	3.81 (2.28, 5.46)	−4.26	<0.001	2.82 (1.60, 4.77)	3.36 (1.77, 4.66)	−0.69	0.492
HOMA2%B	45.4 (30.3, 63.4)	60.3 (37.6, 90.9)	−1.82	<0.001	40.3 (29.2, 64.1)	43.5 (26.7, 91.3)	−0.68	0.493
HOMA2IR	2.03 (1.53, 2.75)	2.23 (1.62, 3.16)	−4.69	0.007	1.64 (1.17, 2.32)	1.86 (1.12, 2.66)	−0.71	0.477

**Note:**

Values are expressed as mean ± standard deviation (SD) or median (range 25th–75th percentile).

### Correlation between SUA and insulin secretory capacity within normal or overweight/obesity groups

The relationship between confounding factors including SUA and insulin secretory capacity within normal or overweight/obesity groups was shown in Table 3. In normal weight group, there were no significant correlations between SUA and insulin secretory capacity. However, in overweight/obesity group, FCP, P2hCP, FCPI, PPCPI, ΔC-peptide, HOMA2%B, and HOMA2IR correlated positively with SUA, while HbA1c correlated negatively with SUA. After adjusting for Cr, BMI, and gender, there were no significant correlations between SUA and HOMA2IR. After additional adjustment for HbA1c and Duration, SUA still had positive significant correlations with insulin secretory capacity include P2hCP, FCPI, PPCPI, ΔC-peptide, and HOMA2%B.

**Table 3:** Correlation of selected variables with SUA in T2DM patients with overweight/obesity group.

	Crude		Adjusted for Cr, BMI, sex		Adjusted for Cr, BMI, sex, HbA1c, Duration	
	*r*	*p*	*r*	*p*	*r*	*p*
HbA1c	−0.186	0.002	−0.226	<0.001		
FCP	0.194	0.001	0.130	0.034	0.115	0.085
P2hCP	0.286	<0.001	0.274	<0.001	0.220	0.001
FCPI	0.268	<0.001	0.222	<0.001	0.142	0.034
PPCPI	0.308	<0.001	0.296	<0.001	0.232	<0.001
ΔC-peptide	0.255	<0.001	0.275	<0.001	0.215	0.001
HOMA2%B	0.257	<0.001	0.235	<0.001	0.137	0.040
HOMA2IR	0.142	0.020	0.082	0.158	0.105	0.117

To further define the relation between SUA and HOMA2%B in overweight/obesity group, multiple linear regression was carried out using SUA as the dependent variable (Table 4). FCP, P2hCP, FCPI, PPCPI, and ΔC-peptide were excluded from the model because of their high correlation with HOMA2%B. FBS and P2hBS were also excluded because of their high correlation with HbA1c. SUA levels were significantly associated with HOMA2%B in unadjusted analyses. After adjustments for sex, Cr, BMI, HbA1c, and Duration, SUA remained positively associated with HOMA2%B.

**Table 4:** Multiple linear regression analysis for SUA and HOMA2%B in T2DM patients with overweight/obesity.

	Partial regression coefficient (B)	Standard error (SE)	Standard partial regression coefficient (β)	*t*	*p*-Value
HOMA2%B (unadjusted)	0.076	0.018	0.257	4.337	<0.001
HOMA2%B (adjusted for model 1: sex, Cr, BMI)	0.066	0.017	0.223	3.930	<0.001
HOMA2%B (adjusted for model 2: model 1, HbA1c and Duration)	0.049	0.022	0.182	2.135	0.013

### Clinical and laboratory data of the patients according to gender and SUA category

The characteristic of the study patients according to gender was shown in Table 5. There were 234 males and 146 females. The male group was younger and had a shorter diabetic duration compared to the female group. Compared with the female group, the levels of SUA, ALT, and CR in the male group were higher. Furthermore, the patients were divided into two groups according to the median SUA levels of patients in the male (LSUA ≤292.0 umol/L; HSUA >292.0 umol/L) or female (LSUA ≤264.5 umol/L; HSUA >264.5 umol/L) group, respectively (Table 6). In the male subgroup, the levels of BMI, ALT, HbA1c, P2hCP, FCPI, PPCPI, ΔC-peptide, and HOMA2%B were higher in HSUA group than in LSUA group. In contrast, the HbA1c, FBS, and P2hBS were lower in HSUA than in LSUA. In the female subgroup, the levels of BMI, TG, CR, and HOMA2IR were higher in HSUA group than in LSUA group.

**Table 5:** Clinical characteristics and islet function indexes of T2DM patients by gender.

Variables	Male (*N* = 234)	Female (*N* = 146)	*t/Z*	*p*
SUA (umol/L)	292.0 (256.0, 339.5)	264.5 (233.5, 297.0)	5.01	<0.001
Age (years)	49.9 ± 12.3	57.2 ± 9.9	−6.03	<0.001
Duration (years)	3.3 (0, 8.0)	5.5 (1.0, 10.0)	−3.38	0.001
SBP (mmHg)	128.0 (114.8, 136.5)	130.0 (118.0, 140.0)	−0.91	0.363
DBP (mmHg)	80.0 (75.5, 90.0)	80.0 (70.0, 84.5)	−3.24	0.001
BMI (kg/m^2^)	25.5 (23.8, 27.7)	25.4 (23.4, 27.3)	−1.71	0.088
TG (mmol/L)	1.99 (1.12, 3.13)	1.54 (0.96, 2.29)	−2.89	0.004
TCH (mmol/L)	4.44 (3.87, 5.20)	4.52 (3.94, 5.15)	−0.32	0.752
LDL (mmol/L)	2.58 (2.09, 3.10)	2.58 (2.34, 3.01)	−1.23	0.220
HDL (mmol/L)	0.95 (0.74, 1.07)	1.07 (0.92, 1.34)	−4.61	<0.001
ALT (U/L)	21.5 (16.0, 35.3)	18.0 (13.0, 26.9)	−3.68	<0.001
CR (umol/L)	75.5 (64.0, 87.3)	67.0 (56.8, 76.6)	−4.89	<0.001
HbA1c (%)	9.22 (7.98, 10.70)	8.45 (7.00, 11.2)	−1.97	0.051
FBS (mmol/L)	9.50 ± 2.99	9.16 ± 3.35	1.04	0.301
P2hBS (mmol/L)	18.85 ± 4.36	18.80 ± 4.81	0.11	0.913
FCP (nmol/L)	2.25 (1.69, 3.27)	2.26 (1.58, 3.03)	−1.33	0.182
P2hCP (nmol/L)	5.53 (3.94, 7.34)	5.74 (4.21, 8.04)	−0.94	0.346
FCPI	0.27 (0.18, 0.36)	0.26 (0.17, 0.35)	−0.43	0.671
PPCPI	1.61 (1.04, 2.45)	1.71 (1.01, 2.85)	−0.91	0.365
ΔC-peptide	2.97 (1.74, 4.30)	3.29 (1.95, 5.40)	−1.97	0.053
HOMA2%B	47.3 (31.6, 75.1)	52.3 (30.5, 79.9)	−0.75	0.471
HOMA2IR	2.04 (1.47, 3.09)	2.02 (1.31, 2.63)	0.45	0.140

**Note:**

Values are expressed as mean ± standard deviation (SD) or median (range 25th–75th percentile).

**Table 6:** Clinical characteristics and islet function indexes of male and female group by the median of SUA.

Variables	Male group (*n* = 234)				Female group (*n* = 146)			
	LSUA <292 umol/L	HSUA ≥292 umol/L	*t/Z*	*p*	LSUA <264.5 umol/L	HSUA ≥264.5umol/L	*t/Z*	*p*
Age (years)	50.78 ± 12.79	48.91 ± 11.76	1.16	0.247	55.90 ± 9.27	58.3 ± 10.49	−1.44	0.153
Duration (years)	4.0 (0.1, 8.0)	3.0 (0.0, 7.0)	−0.76	0.447	5.0 (1.0, 10.0)	6.0 (1.3, 10.0)	−0.70	0.481
SBP (mmHg)	128.0 (115.0, 136.0)	126.0 (114.0, 138.0)	−0.35	0.726	128.0 (118.0, 136.0)	130.0 (120.0, 140.0)	−1.26	0.209
DBP (mmHg)	80.0 (74.0, 90.0)	80.0 (76.0, 90.0)	−0.45	0.685	76.0 (70.0, 80.0)	80.0 (70.0, 88.0)	−1.20	0.229
BMI (kg/m^2^)	25.4 (23.2, 26.6)	25.9 (24.5, 28.4)	−3.44	0.001	24.9 (22.5, 26.1)	25.5 (23.6, 27.9)	−2.02	0.044
TG (mmol/L)	1.93 (1.02, 2.59)	2.01 (1.27, 3.35)	−1.69	0.089	1.43 (0.91, 1.91)	1.82 (1.03, 2.72)	−2.21	0.027
TCH (mmol/L)	4.38 (3.80, 5.19)	4.57 (3.92, 5.18)	−0.99	0.319	4.55 (3.95, 5.22)	4.43 (3.91, 5.05)	−0.55	0.585
LDL (mmol/L)	2.58 (2.07, 3.10)	2.58 (2.15, 3.12)	−0.88	0.380	2.58 (2.33, 2.99)	2.58 (2.34, 3.02)	−0.36	0.720
HDL (mmol/L)	0.98 (0.74, 1.10)	0.91 (0.76, 1.07)	−0.85	0.395	1.10 (1.01, 1.63)	1.07 (0.81, 1.23)	−3.38	0.001
ALT (U/L)	18.0 (14.0, 31.0)	25.0 (18.0, 42.0)	−3.48	<0.001	19.0 (13.0, 26.5)	18.0 (13.5, 27.0)	−0.42	0.676
CR (umol/L)	76.0 (63.0, 88.0)	74.0 (65.0, 86.0)	−0.03	0.978	62.0 (52.5, 74.0)	72.0 (61.5, 78.5)	−2.95	0.003
HbA1c (%)	9.90 (8.30, 11.30)	8.80 (7.70, 10.0)	4.30	<0.001	8.10 (6.85, 10.65)	8.73 (7.02, 11.42)	−1.18	0.237
FBS (mmol/L)	10.09 ± 2.82	8.89 ± 3.05	3.10	0.002	8.75 ± 3.17	9.57 ± 3.49	−1.50	0.137
P2hBS (mmol/L)	19.78 ± 3.99	17.88 ± 4.55	3.42	0.001	18.29 ± 4.76	19.32 ± 4.82	−1.33	0.186
FCP (nmol/L)	2.20 (1.65, 2.91)	2.46 (1.73, 3.60)	−1.95	0.051	2.11 (1.29, 2.85)	2.40 (1.78, 3.23)	−1.99	0.046
P2hCP (nmol/L)	4.85 (3.54, 6.67)	6.19 (4.48, 8.06)	−3.95	<0.001	5.41 (3.79, 7.76)	6.17 (4.45, 8.65)	−1.48	0.138
FCPI	0.22 (0.17, 0.33)	0.31 (0.22, 0.47)	−3.93	<0.001	0.25 (0.16, 0.33)	0.26 (0.17, 0.41)	−0.86	0.392
PPCPI	1.35 (0.92, 1.96)	2.03 (1.41, 2.69)	−4.53	<0.001	1.69 (1.05, 2.76)	1.84 (0.99, 3.08)	−0.91	0.362
ΔC-peptide	2.32 (1.38, 3.84)	3.55 (2.26, 5.27)	−4.01	<0.001	3.08 (1.84, 5.25)	3.75 (2.19, 5.51)	−1.06	0.288
HOMA2%B	38.3 (27.9, 59.8)	59.3 (37.3, 89.0)	−4.39	<0.001	56.5 (37.2, 74.6)	48.7 (27.2, 86.7)	−0.27	0.784
HOMA2IR	1.98 (1.49, 2.65)	2.20 (1.43, 3.25)	−1.24	0.214	1.81 (1.19, 2.49)	2.18 (1.54, 2.97)	−2.38	0.017

**Note:**

Values are expressed as mean ±standard deviation (SD) or median (range 25th–75th percentile).

### Correlation between SUA and insulin secretory capacity by gender category

The relationship between confounding factors including SUA and insulin secretory capacity within male or female groups was shown in Table 7. In male group, FCP, P2hCP, FCPI, PPCPI, ΔC-peptide, and HOMA2%B correlated positively with SUA, while HbA1c correlated negatively with SUA. After adjusting for Cr and BMI, there were also significant correlations between SUA and HOMA2IR. After additional adjustment for HbA1c and Duration, SUA still had positive significant correlations with insulin secretory capacity include P2hCP, FCPI, PPCPI, ΔC-peptide, and HOMA2%B. However, in female group, SUA only correlated positively with P2hCP and ΔC-peptide.

**Table 7:** Correlation of SUA with selected variables in T2DM patients with male and female group.

		Crude		Adjusted for Cr, BMI		Adjusted for Cr, BMI, HbA1c, Duration	
		*r*	*p*	*r*	*p*	*r*	*p*
Male group	HbA1c	−0.291	<0.001	−0.284	<0.001		
	FCP	0.235	0.001	0.142	0.031	0.101	0.127
	P2hCP	0.331	<0.001	0.280	<0.001	0.163	0.013
	FCPI	0.356	<0.001	0.288	<0.001	0.178	0.007
	PPCPI	0.351	<0.001	0.322	<0.001	0.195	0.003
	ΔC-peptide	0.293	<0.001	0.273	<0.001	0.147	0.026
	HOMA2%B	0.350	<0.001	0.319	<0.001	0.195	0.003
	HOMA2IR	0.156	0.017	0.065	0.322	0.066	0.320
Female group	HbA1c	0.013	0.876	0.011	0.884		
	FCP	0.165	0.046	0.108	0.199	0.113	0.182
	P2hCP	0.203	0.014	0.182	0.029	0.200	0.017
	FCPI	0.171	0.039	0.137	0.101	0.155	0.066
	PPCPI	0.135	0.104	0.114	0.175	0.133	0.116
	ΔC-peptide	0.182	0.028	0.177	0.034	0.198	0.018
	HOMA2%B	0.134	0.106	0.135	0.108	0.163	0.053
	HOMA2IR	0.149	0.072	0.090	0.282	0.094	0.268

To further define the relation between SUA and HOMA2%B or HOMA2IR, multiple linear regression was carried out using SUA as the dependent variable (Table 8). In male group, SUA levels were significantly associated with HOMA2%B in unadjusted analyses. After adjustments for Cr, BMI, HbA1c and Duration, SUA remained positively associated with HOMA2%B. SUA levels were significantly associated with HOMA2IR in unadjusted analyses. After adjustments for Cr, BMI, HbA1c and Duration, there were no significant correlations between SUA and HOMA2IR. In contrast, there were no significant correlations between SUA and HOMA2%B and HOMA2IR in the female group.

**Table 8:** Multiple linear regression analysis for SUA and HOMA2%B or HOMA2IR in T2DM patients by gender category.

		Partial regression coefficient (B)	Standard error (SE)	Standard partial regression coefficient (β)	*t*	*p*-Value
Male group	HOMA2%B					
	Unadjusted	0.514	0.090	0.350	5.69	<0.001
	Adjusted for model 1: Cr, BMI	0.458	0.090	0.312	5.10	<0.001
	Adjusted for model 2: model 1, HbA1c and Duration	0.319	0.107	0.217	2.99	0.003
	HOMA2IR					
	Unadjusted	2.986	3.323	0.156	2.40	0.017
	Adjusted for model 1: Cr, BMI	3.415	3.443	0.067	0.99	0.322
	Adjusted for model 2: model 1, HbA1c and Duration	3.346	3.359	0.065	0.99	0.320
Female group	HOMA2%B					
	Unadjusted	0.141	0.087	0.134	1.626	0.106
	Adjusted for model 1: Cr, BMI	0.137	0.085	0.131	1.618	0.108
	Adjusted for model 2: model 1, HbA1c and Duration	0.197	0.101	0.188	1.949	0.053
	HOMA2IR					
	Unadjusted	4.703	2.593	0.149	1.814	0.072
	Adjusted for model 1: Cr, BMI	2.783	2.578	0.088	1.079	0.282
	Adjusted for model 2: model 1, HbA1c and Duration	2.940	2.646	0.093	1.111	0.268

### Correlation between islet function/insulin resistance and related variables in T2DM patients

To identify confounding factors affecting islet function and insulin resistance, a multiple linear regression was again performed in T2DM patients. Independent variables such as SUA, age, gender, diabetic duration, SBP, DBP, BMI, TG, TCH, LDL, HDL, ALT, CR, HbA1c were enrolled (Table 9). HOMA2%B had positive associations with BMI, SUA, age and diabetic duration and a negative correlation with HbA1c. HOMA2IR had positive associations with BMI and TG and a negative correlation with diabetic duration.

**Table 9:** Multiple linear regression analysis on related variables for islet function indexes in T2DM patients.

		Partial regression coefficient (B)	Standard error (SE)	Standard partial regression coefficient (β)	*p*-Value
HOMA2%B	HbA1c	−9.103	0.781	−0.501	<0.001
	SUA	0.127	0.032	0.177	<0.001
	Age	0.486	0.159	0.146	0.002
	BMI	1.143	0.522	0.095	0.029
	Duration	−0.697	0.327	−0.100	0.034
HOMA2IR	BMI	0.089	0.018	0.241	<0.001
	TG	0.076	0.023	0.165	0.001
	Duration	−0.029	0.010	−0.134	0.006

## Discussion

In this study, we confirmed that SUA levels are significantly associated with HOMA2%B in T2DM patients with overweight/obesity and male group, but not in normal weight and female group. In addition, we also demonstrated that other islet function indexes, such as FCPI, PPCPI, and ΔC-peptide, did correlate with SUA levels in T2DM patients with overweight/obesity and male group. However, our study observed the absence of a relationship between SUA and HOMA2IR after adjustment for Cr, BMI, sex, HbA1c, and diabetic duration in T2DM patients with overweight/obesity or male. To the best of our knowledge, this study is the first that these effects of SUA within the normal range on determinants of β-cell function and insulin resistance in T2DM by BMI and gender categories.

Uric acid is the end product of purine metabolism and derives from the conversion of hypoxanthine to xanthine and of xanthine to uric acid. We observed that SUA was higher in T2DM patients with overweight/obesity group than in those with normal weight group, SUA within normal range independently related to obesity in T2DM. Consistent with our results, several previous studies have also shown the relationship between BMI and uric acid (Han et al., 2018). For example, Chen et al. (2017) also found that prevalence of obesity steadily increased across SUA quartiles in T2DM. A 10-year follow-up study demonstrated that BMI had a significant independent association with uric acid in all race-sex-groups (Rathmann et al., 2007). Furthermore, in subjects without diabetes or hyperuricemia, SUA levels were also associated with BMI, waist circumference, and waist-to-hip ratio (Jin et al., 2013). Interestingly, Zhou et al. (2017) found that successful weight control, mostly >10 kg weight reduction, was correlated with significant uric acid reduction after 2 years observation. Therefore, SUA levels, even in normal range, were associated with BMI in T2DM patient.

In addition to strong association with BMI, SUA is also associated with β-cell function in T2DM. Tang et al. (2014) found that patients with higher levels of SUA had higher insulin secretion, including the early phase and total insulin secretion in T2DM patients. Similarly, another study (Hu et al., 2018) has also reported that SUA augments insulin secretion, particularly basal insulin secretion, in the population-based study of newly diagnosed T2DM. Even in nondiabetic population, higher SUA levels also significantly correlate with lower early-phase insulin secretion (Shimodaira et al., 2014). However, the abovementioned studies do not evaluate the relationship between SUA in the normal range and β-cell function. Most of prior studies researching the association between SUA and β-cell function did not conduct subgroup analyses by BMI categories. Our present results show that SUA in the normal range is significantly associated with HOMA2%B in T2DM patients with overweight/obesity, but not in the normal weight group. Although it is not possible to explain the mechanism underlying this body weight difference from our study, this observation may be due to the influence of SUA levels, which our study showed that SUA levels were higher in T2DM patients with overweight/obesity than in those with normal weight group. Although subjects with higher SUA secrete more insulin, it does not mean that high SUA is beneficial to β-cell function. SUA becomes a strong oxidant in the environment of obesity (Johnson et al., 2009), which may in turn promote lipid oxidation. In addition, obesity is related to elevated SUA level via both low urinary urate excretion and overproduction of SUA (Matsuura et al., 1998). A recent study found that an elevated level of uric acid causes β-cell injury via the NFκB-iNOS-NO signaling axis (Jia et al., 2013). Furthermore, Sun et al. (2015) found that uric acid-associated genes have an impact on insulin secretion in a Chinese patients with T2DM. Finally, another study (Seyed-Sadjadi et al., 2017) showed that the associations between SUA and diabetes risk factors are largely dependent on visceral fat mass in a non-diabetic population. Physicochemical properties define hyperuricemia as levels above the solubility threshold (6.8 mg/dL). With regard to metabolic sequel, high-normal SUA levels are already associated with an increased risk in patient with overweight/obesity.

The disposition index (DI) is thought to reflect the capacity for insulin secretion adjusted for insulin sensitivity and thus to provide a useful measure of β-cell function. PP-CPI, a ratio of the circulating level of C-peptide to that of glucose, is correlated with clamp DI (Okuno et al., 2013). In the present study, we found that PPCPI and ΔC-peptide had positive associations with SUA levels in overweight/obesity group, but not in normal weight group. Our findings agree with previous report by Tang et al. (2014), which shows that patients with higher SUA had greater disposition indices (both DI30 and DI120). Taken together, accumulated evidence suggest SUA levels may be associated with insulin secretion in T2DM patients with overweight/obesity.

Another important finding in our study was that SUA had positive significant correlations with insulin secretory capacity include P2hCP, FCPI, PPCPI, ΔC-peptide, and HOMA2%B in male group. Hyperuricemia affected men more commonly than women. There was a SUA difference of 30–120 umol/L between men and women (Akizuki, 1982). It is previously known that estrogen may promote excretion of uric acid (Hu et al., 2018). Together, these result indicate that gender differences in association between SUA within normal range and insulin secretion in patients with T2DM. However, a previous study (Hu et al., 2018) suggested that elevated SUA was associated with insulin secretion in male and female. The mechanism underlying this sex-based difference remains unclear, and requires further study.

The evidence of the linkage between SUA and insulin resistance in type 2 diabetes is growing, but it is unclear if SUA within the normal range directly lead to declines in insulin sensitivity in T2DM patients. However, our study observed the absence of a relationship between SUA within normal range and insulin resistance in T2DM patients with overweight/obesity and normal weight groups. Other researchers (Hu et al., 2018; Wang et al., 2011) have also demonstrated that the UA levels of hyperuricemic patients have no effect on their insulin sensitivity index. Liu & Ho (2011) study suggested that SUA was not associated with insulin resistance after adjustment for BMI, TG, and BP. There are several possible explanations for the lack of independent relationship between SUA within normal range and insulin resistance in this study. Firstly, this result could be driven by SUA levels that are well within the normal range. Secondly, these discrepancies could be related the techniques used for measurement of insulin sensitivity. Finally, UA has an important role as an antioxidant (Lippi et al., 2008), but elevated SUA may cause oxidative stress (Pasalic, Marinkovic & Feher-Turkovic, 2012) and inhibit endothelial NO bioavailability (Sharaf El Din, Salem & Abdulazim, 2017), all of which closely associated with the insulin resistance. Collectively, the exact role of SUA within normal range in oxidation is still worth further investigation in T2DM patients.

The relationship between SUA and HbA1c has been reported. For example, Kawamoto et al. (2018) found a negative association between SUA and HbA1c was shown particularly in men with HbA1c ≥6.5%. Cui et al. (2016) showed that a negative correlation between uric acid and HbA1c is conditional in newly diagnosed type 2 diabetes patients. In our study, we also found that SUA within normal range negatively related to HbA1c in T2DM patients with overweight/obesity. In T2DM patients with normal weight group, the partial correlation analysis demonstrated the negative correlation between SUA and HbA1c, but no significant difference was observed with multiple linear regression analysis. These results indicated that there was negatively association between SUA, even within normal range, and HbA1c in T2DM patients with overweight/obesity.

Unfortunately, this study has some limitations. Firstly, we do not analyze whether oral hypoglycemic agents have an effect on SUA. Sodium-glucose co-transporter 2 inhibitor (SGLT-2i) could improve glycemic control and lower SUA levels in T2DM (Hao et al., 2018). However, other hypoglycemic drugs, including metformin, rosiglitazone, glibenclamide, and pharmacologic insulin, do not have a large impact on SUA concentration (Hussain et al., 2018; Iliadis et al., 2007; MacFarlane, Liu & Solomon, 2015). In our study, the T2DM patients were treated with oral hypoglycemic drugs (not including SGLT-2i) and insulin. Secondly, the number of subjects enrolled was relatively small. Thirdly, the relationship between SUA within normal range and oxidative stress is still worth further investigation in T2DM.

## conclusion

Our study shows that SUA levels within normal range are associated with β-cell function in T2DM patients with overweight/obesity, and the relationship also displays sex-based differences. However, SUA levels within normal range are not related to insulin resistance in T2DM patients. This finding supports the association between SUA within normal range and insulin secretion ability differs by weight and gender.

## Supplemental Information

10.7717/peerj.6666/supp-1 Supplemental Information 1 Clinical characteristicsand insulin secretory capacity and insulin resistance index of all participants with T2DM. Raw data showed age, gender, diabetic duration, blood pressure, blood lipid profiles, renal function, HbA1c, body mass index, insulin secretory capacity and insulin resistance index of all the participants .

## Additional Information and Declarations

### Data Availability

The following information was supplied regarding data availability: The raw data is available as a Supplemental File.
